# Burden of gallbladder and biliary tract cancer (GBTC) in the middle east and North Africa (MENA) from 1990 to 2021: from the global burden of disease (GBD)

**DOI:** 10.1097/MS9.0000000000005178

**Published:** 2026-07-03

**Authors:** Babak Mansourian, Tarannom Sadegh, Amirhossein Habibzadeh, Sogol Hajifathali, Asal Khalili Dehkordi, Mahshid Jamshidi, Mohammad Sharifi Sarasyabi, Kasra Hatampour, Khashayar Danandeh

**Affiliations:** a Department of General Surgery, Shahid Madani Clinical Research Development Unit, Student Research Committee, School of Medicine, Alborz University of Medical Sciences, Karaj, Iran; b Department of Computer Engineering, University of Science and Culture, Tehran, Iran; c Department of Medicine, School of Medicine, Tehran University of Medical Sciences, Tehran, Iran; d Department of Hematology, Hematopoietic Stem Cell Research Center, Shahid Beheshti University of Medical Sciences, Tehran, Iran; e Department of Cardiovascular Surgery, Research Center for Advanced Technologies in Cardiovascular Medicine, Cardiovascular Research Institute, Tehran University of Medical Sciences, Tehran, Iran; f Department of Pediatrics, Growth and Development Research Center, Children’s Medical Center, Tehran University of Medical Sciences, Tehran, Iran; g Department of Neurosurgery, Functional Neurosurgery Research Center, Research Institute of Functional Neurosurgery, Shohada Tajrish Comprehensive Neurosurgical Center of Excellence, Shahid Beheshti University of Medical Sciences, Tehran, Iran; h Department of Medicine, Medical Informatics Research Center, Institute for Futures Studies in Health, Kerman University of Medical Sciences, Kerman, Iran; i Department of General Surgery, Loghman Medical Center, Shahid Beheshti University of Medical Sciences, Tehran, Iran; j Neuromusculoskeletal Research Center, Department of Physical Medicine and Rehabilitation, School of Medicine, Iran University of Medical Sciences, Tehran, Iran

**Keywords:** cancer, gallbladder and biliary tract cancer, global burden of disease, MENA, public health

## Abstract

**Introduction:**

Gallbladder and biliary tract cancer (GBTC) is a rare but aggressive malignancy with poor survival outcomes, mainly due to late-stage diagnosis and limited treatment options. Despite prominent global disparities in incidence and outcomes, comprehensive data on the GBTC burden in the Middle East and North Africa (MENA) region remain scarce.

**Materials and methods:**

We used Global Burden of Disease (GBD) 2021 data. We retrieved incidence, deaths, and DALYs, including crude and age-standardized rates per 100 000, with 95% uncertainty intervals for 21 MENA countries, the MENA region, and globally, stratified by year (1990–2021), sex, and age group.

**Results:**

In the MENA region, the age-standardized incidence rate (ASIR) remained at 1.4 per 100 000 in both 1990 and 2021 (−0.2% change), while globally it declined from 2.89 to 2.56 (−11.5%; 95% UI: −21.9 to −3.4). The age-standardized death rate (ASDR) decreased from 1.50 to 1.37 (−9.0%) in MENA versus 2.69 to 2.04 (−24.1%; 95% UI: −33.2 to −16.9) globally. The age-standardized DALY rate fell from 34.26 to 29.82 (−13.0%) in MENA and from 58.58 to 43.20 (−26.2%; 95% UI: −35.5 to −18.4) globally. Across years, females had higher age-standardized rates than males, rates increased with age, and the peak DALY rate shifted from 80–84 (1990) to 85–89 (2021).

**Conclusions:**

While the burden of GBTC has decreased in the MENA region and has consistently remained lower than the global average, there is considerable heterogeneity across countries in the region that warrants the attention of policymakers, particularly in countries with a higher burden of GBTC, such as Libya and the United Arab Emirates, and in countries with an increasing burden, such as Iran.

## Introduction

Gallbladder and biliary tract cancer (GBTC) encompasses a group of invasive malignancies, predominantly adenocarcinomas, originating from the gallbladder or cystic duct and the biliary tract^[^1,2^]^. Although these malignancies are relatively uncommon, they pose a substantial and growing public health challenge due to their aggressive behavior. In 2021, there were an estimated 216 768 new cases and 171 961 deaths worldwide related to GBTC, with global incidence and mortality rates of approximately 2.6 and 2.0 per 100 000 persons, respectively^[^3–5^]^.

These tumors account for only 4% of all cancers of the gastrointestinal system^[^3^]^, and although relatively rare, GBTCs are associated with poor clinical outcomes^[^6^]^. Currently, the primary curative treatment is surgical resection; however, most patients receive their diagnosis at an advanced stage, rendering them ineligible for surgery^[^7^]^. The median survival for all patients with GBTC is about 36.8 months, primarily due to delayed diagnoses resulting from nonspecific clinical presentations and a lack of effective screening methods^[^8^]^.

Significant health disparities in GBTC outcomes exist across sex, age, and socioeconomic status^[^6^]^. Incidence and mortality rates are higher in females and individuals over 55 years old. Moreover, those with lower socioeconomic status often face poorer outcomes, mainly due to limited healthcare access, delays in diagnosis, and increased exposure to behavioral risk factors^[^9^]^. Beyond individual factors, GBTC shows significant geographic, racial, and ethnic disparities, with notable variation in burden across countries and regions^[^3^]^. In the Middle East and North Africa (MENA) region, however, comprehensive and specific Global Burden of Disease (GBD) data on GBTC are largely unavailable in the current literature. Therefore, this study evaluated the burden of GBTC in the MENA region, utilizing data from the GBD 2021 study conducted by the Institute for Health Metrics and Evaluation (IHME)^[^10^]^. This study provided estimates of the burden of GBTC from 1990 to 2021, categorized by region, sex, age, and socio-demographic status. Additionally, this study has been reported in line with the STROCSS guidelines^[^11^]^.

HIGHLIGHTS

- Stable but Uneven Trends: The overall age-standardized incidence of gallbladder and biliary tract cancer (GBTC) remained stable in the MENA region from 1990 to 2021, in contrast to global declines.
- Country-Level Disparities: Libya and the United Arab Emirates showed persistently high burdens, while Iran experienced the most pronounced increases.
- Sex and Age Patterns: Females consistently exhibited higher incidence, mortality, and DALY rates, with the peak burden shifting to older age groups over time.
- Socio-demographic Influence: DALY rates were highest in middle-SDI countries, indicating a nonlinear relationship between development level and disease burden.
- Policy Relevance: Findings highlight the need for country-specific prevention strategies, with an emphasis on early detection and risk factor reduction in high-burden and rising-burden nations.

## Methods and materials

### Overview and data sources

We utilized the GBD 2021 data in this study, which was conducted by the IHME, to assess the burden of 371 diseases and injuries across 204 countries and territories. The GBD 2021 employed standardized methodologies and incorporated updated data sources to ensure comparability of estimates^[^12^]^. We extracted data specific to GBTC from the GBD results tool for the years 1990 through 2021 (https://vizhub.healthdata.org/gbd-results/). This included data at the national level for all countries in the MENA region (which includes Afghanistan, Algeria, Bahrain, Egypt, Iran, Iraq, Jordan, Kuwait, Lebanon, Libya, Morocco, Oman, Palestine, Qatar, Saudi Arabia, Sudan, Syria, Tunisia, Turkey, United Arab Emirates [UAE], and Yemen), as well as aggregate estimates for the entire MENA region and global estimates for comparison (Supplemental Digital Content Table 1, available at: http://links.lww.com/MS9/B271)^[^3,12^]^. As the analyses were based on de-identified and publicly available data, no ethical approval was required.

### Case definition

In GBD 2021, GBTC is defined as malignant neoplasms of the gallbladder and the extrahepatic bile ducts, classified under the International Classification of Diseases, 10th Revision (ICD-10) codes C23 and C24 (including C24.0–C24.9)^[^12^]^. Notably, this definition excludes intrahepatic bile duct carcinoma (ICD-10 code C22.1, a form of cholangiocarcinoma) to avoid overlap with liver cancer in the GBD cause hierarchy. All data for incidence, mortality, and other metrics of GBTC were derived according to this case definition^[^12,13^]^.

### Indices and measurements

We obtained comprehensive epidemiological metrics for GBTC, including incidence, deaths (mortality), and disability-adjusted life years (DALYs), for each MENA country, the MENA region, and globally from 1990 to 2021^[^3,13^]^. For each metric, we collected the absolute number of events, the crude rate per 100 000 population, and the age-standardized rate (ASR) per 100 000 population. Years of life lost (YLL) represent the number of YLL due to premature mortality from GBTC, whereas years lived with disability (YLD) represent the years lived with the disease in less than full health. DALY is a composite measure calculated as the sum of YLL and YLD, thereby quantifying the total health loss due to the condition. Age-standardized rates were computed using the GBD standard population to enable comparisons across populations and over time^[^13^]^. Data were retrieved for both sexes, across all age groups, and were accompanied by 95% uncertainty intervals (UIs). In the GBD study framework, each reported measure is derived from 1000 statistical draws; the mean of these draws is taken as the point estimate, and the 2.5th and 97.5th percentiles form the bounds of the 95% UI.

### Statistical analyses

We performed all data management and analyses using Python (version 3.10.12). Visualization and geographical mapping of results were conducted in Python with libraries including NumPy, Pandas, GeoPandas, Matplotlib, and Seaborn. We used locally estimated scatterplot smoothing (LOESS) regression (smoothing parameter = 0.3) to evaluate the burden of GBTC, as determined by its age-standardized DALY rate across MENA countries and years, and SDI.

## Results

### Overall trends in incidence, mortality, and DALYs

**Figure 1.**
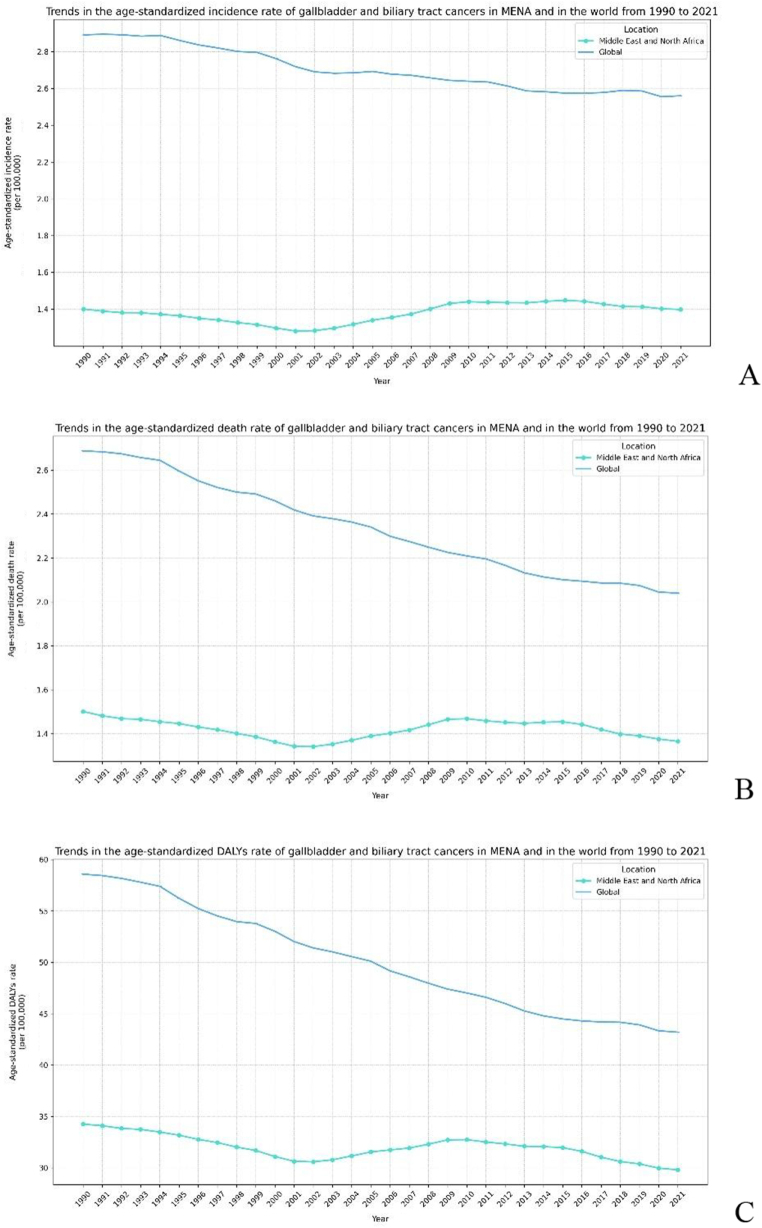
Age-standardized incidence (A), death (B), and DALY (C) rates of gallbladder and biliary tract cancers in MENA and the world, 1990–2021.

**Figure 2.**
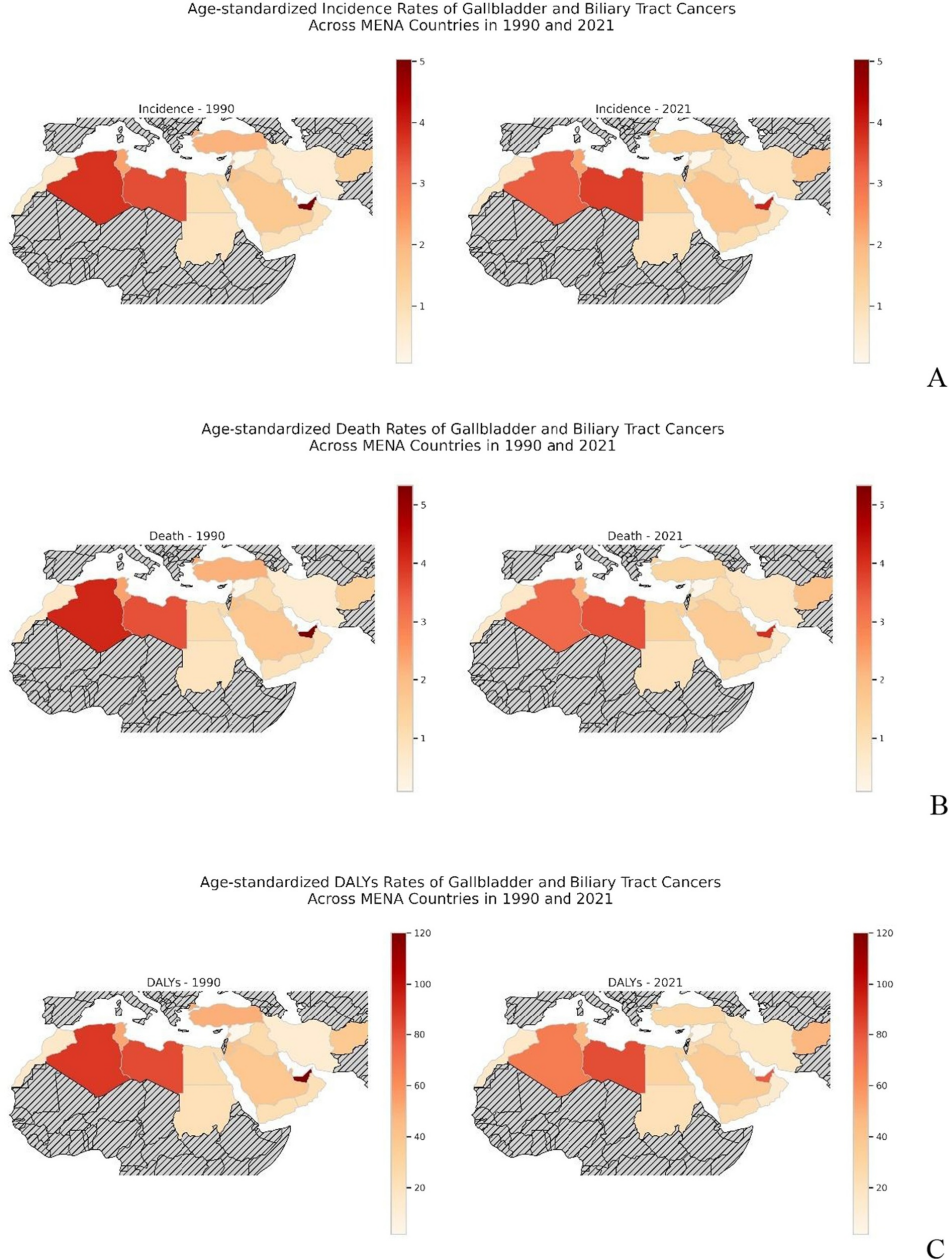
Age-standardized incidence (A), death (B), and DALY (C) rate of gallbladder and biliary tract cancers across MENA countries in 1990 and 2021.

In the MENA region, the age-standardized incidence rate (ASIR) of GBTC remained almost stable between 1990 and 2021, at 1.4 per 100 000 population, with a negligible change of 0.2%. In contrast, the global ASIR declined by 11.5%, from 2.89 in 1990 to 2.56 per 100 000 in 2021 (Table 1, Fig. 1). Among MENA countries, 10 showed an increase in ASIR, and 11 showed a decrease. The UAE had the highest ASIR in 2021 at 3.95 per 100 000, followed by Libya (3.60 per 100 000) and Algeria (3.23 per 100 000). The lowest ASIR values were recorded in Syria (0.09 per 100 000), Morocco (0.67 per 100 000), and Oman (0.75 per 100 000) (Fig. 2). The largest increase was observed in Iran, where ASIR rose by 69.0% (from 0.52 to 0.88 per 100 000), while Kuwait showed the greatest decline at 29.7% (from 1.86 to 1.31 per 100 000) (Table 1).

**Table 1:** Incidence, death, and DALYs rates of gallbladder and biliary tract cancers across Middle East and North Africa countries and the world in 1990 and 2021 for.

Location	Age-standardized incidence rate			Age-standardized death rate			Age-standardized DALY rate		
	1990	2021	% Change	1990	2021	% Change	1990	2021	% Change
World	2.89	2.56	−11.5 (−21.9, −3.4)	2.69	2.04	−24.1 (−33.2, −16.9)	58.58	43.20	−26.2 (−35.5, −18.4)
Middle East and North Africa	1.40	1.40	−0.2 (−16.7, 17.4)	1.50	1.37	−9.0 (−24.2, 6.7)	34.26	29.82	−13.0 (−26.7, 3.0)
Afghanistan	1.37	1.78	29.4 (−17.3, 94.3)	1.46	1.90	29.8 (−17.0, 91.8)	37.67	47.15	25.2 (−20.7, 92.9)
Algeria	3.77	3.23	−14.2 (−31.8, 8.2)	4.12	3.28	−20.4 (−35.9, −0.2)	88.09	67.42	−23.5 (−38.9, −2.7)
Bahrain	1.14	0.99	−13.1 (−35.9, 16.9)	1.22	0.92	−24.5 (−43.9, 0.9)	25.48	17.91	−29.7 (−48.7, −3.4)
Egypt	1.02	1.37	34.8 (−10.8, 74.9)	1.10	1.42	28.2 (−15.8, 66.1)	25.74	31.06	20.7 (−16.3, 58.0)
Iran	0.52	0.88	69.0 (2.2, 116.9)	0.55	0.82	49.9 (−8.4, 91.8)	12.28	17.86	45.5 (−12.2, 89.5)
Iraq	1.03	1.07	4.3 (−27.1, 44.2)	1.07	1.06	−1.4 (−32.3, 35.8)	26.71	23.82	−10.8 (−37.1, 25.0)
Jordan	1.74	1.29	−25.7 (−47.7, 9.1)	1.83	1.22	−33.6 (−52.6, −2.8)	41.90	26.06	−37.8 (−57.1, −6.8)
Kuwait	1.86	1.31	−29.7 (−41.5, −15.8)	1.76	1.04	−41.2 (−51.2, −28.8)	38.85	21.74	−44.0 (−53.8, −32.2)
Lebanon	1.87	1.56	−16.3 (−42.0, 35.7)	1.96	1.40	−28.5 (−50.1, 16.4)	43.11	28.56	−33.7 (−54.0, 10.6)
Libya	3.44	3.60	4.7 (−25.7, 45.4)	3.61	3.58	−0.6 (−28.8, 39.1)	82.78	81.81	−1.2 (−30.1, 38.9)
Morocco	0.63	0.67	5.9 (−20.5, 36.3)	0.68	0.69	2.3 (−23.4, 31.8)	15.87	15.71	−1.0 (−26.4, 31.0)
Oman	0.92	0.75	−18.9 (−40.7, 16.1)	0.96	0.68	−29.2 (−48.0, −0.4)	22.04	14.58	−33.9 (−52.0, −4.0)
Palestine	1.62	1.34	−17.3 (−37.6, 12.5)	1.74	1.34	−22.8 (−41.9, 5.8)	37.82	28.29	−25.2 (−45.4, 4.6)
Qatar	2.15	1.68	−21.8 (−44.2, 7.6)	2.27	1.39	−38.6 (−56.1, −15.7)	45.47	26.91	−40.8 (−58.0, −16.6)
Saudi Arabia	1.60	1.72	7.5 (−19.2, 48.3)	1.72	1.60	−7.0 (−30.0, 27.4)	38.77	36.24	−6.5 (−30.5, 33.2)
Sudan	0.83	0.88	6.9 (−45.0, 93.4)	0.88	0.93	4.8 (−45.2, 89.2)	21.77	21.94	0.8 (−47.4, 85.3)
Syria	0.07	0.09	18.7 (−25.6, 78.4)	0.08	0.09	8.5 (−32.0, 62.1)	1.82	1.87	2.7 (−35.4, 58.7)
Tunisia	2.28	2.27	−0.7 (−28.7, 38.7)	2.41	2.13	−11.3 (−36.4, 23.0)	52.61	46.30	−12.0 (−37.6, 26.8)
Turkey	2.04	1.46	−28.4 (−45.7, −1.6)	2.19	1.36	−37.8 (−52.6, −13.6)	50.11	29.17	−41.8 (−56.6, −19.6)
UAE	5.03	3.95	−21.4 (−41.0, 21.7)	5.33	4.04	−24.3 (−43.1, 15.1)	120.17	78.07	−35.0 (−51.7, 1.0)
Yemen	0.88	0.99	12.8 (−34.0, 82.9)	0.95	1.06	11.9 (−33.9, 80.9)	22.68	24.71	8.9 (−36.5, 84.2)

The age-standardized death rate (ASDR) of GBTC in the MENA region decreased from 1.50 per 100 000 in 1990 to 1.37 per 100 000 in 2021, representing a 9.0% reduction. The global change in ASDR over the same period was − 24.1%, from 2.69 to 2.04 per 100 000 (Fig. 1). Of all MENA countries, 14 experienced a decrease in ASDR, while 7 showed an increase. The UAE had the highest ASDR in MENA in 2021 (4.04 per 100 000), followed by Libya (3.58 per 100 000) and Algeria (3.28 per 100 000), while Syria (0.09 per 100 000), Oman (0.68 per 100 000), and Morocco (0.69 per 100 000) had the lowest (Fig. 2). The largest increase in ASDR was reported in Iran (49.9%), and the most significant decline occurred in Kuwait (−41.2%) (Table 1).

The age-standardized DALY of GBTC rate in MENA declined by 13.0% over the study period, from 34.26 in 1990 to 29.82 per 100 000 in 2021. The global DALY rate changed by −26.2%, from 58.58 to 43.20 per 100 000 (Fig. 1). DALY rates decreased in 15 MENA countries and increased in 6. The highest DALY rates in 2021 were recorded in Libya (81.81 per 100 000), the UAE (78.07 per 100 000), and Algeria (67.42 per 100 000), while the lowest were in Syria (1.87 per 100 000), Oman (14.58 per 100 000), and Morocco (15.71 per 100 000) (Fig. 2). Iran experienced the greatest increase (45.5%), whereas Kuwait (−44.0%), Turkey (−41.8%), and Qatar (−40.8%) recorded the most pronounced declines (Table 1). DALY rates are age-standardized per 100 000.

### Sex and age-specific patterns

**Figure 3.**
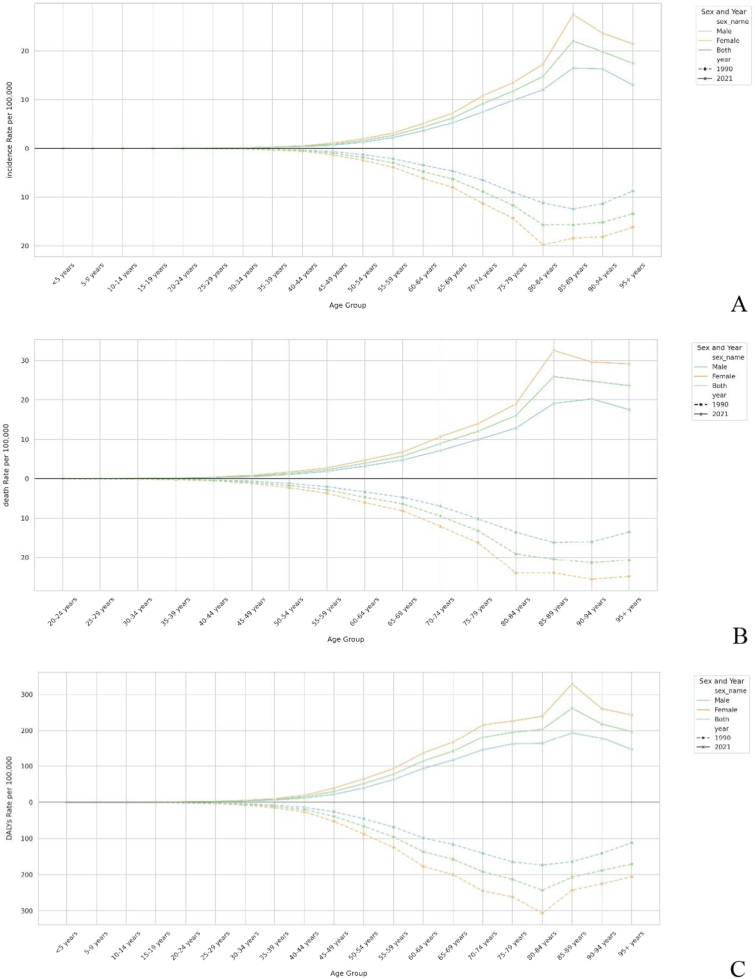
Incidence (A), death (B), and DALY (C) rate of gallbladder and biliary tract cancers by sex and age group in 1990 and 2021 in MENA.

Throughout the study period, females consistently experienced a higher burden of GBTC than males with regard to incidence, death, and DALY rates (Fig. 3). This pattern was evident across most age groups and persisted from 1990 to 2021. The incidence, death, and DALY rates of GBTC were the highest in the 85–89 years age group in 2021, while the incidence and DALY rates were the highest in the 80–84 years age group, and the death rate was highest in the 90–94 years age group in 1990 (Fig. 3).

### SDI and DALY rate associations

**Figure 4.**
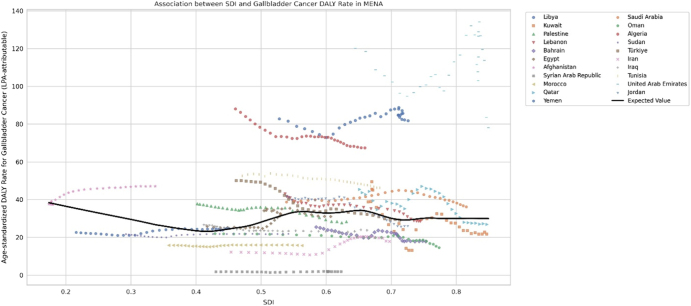
Association between the age-standardized DALY rate of gallbladder and biliary tract cancers and the sociodemographic index across MENA countries and years.

Figure 4 illustrates the relationship between SDI and the age-standardized DALY rate of GBTC across MENA countries and years, indicating a non-linear association. The highest age-standardized DALY rate occurred at around the SDI level of 0.15, then fell to a low point at approximately an SDI of 0.4, after which it began to rise again and, despite some fluctuations, remained high.

## Discussion

The results of this study demonstrated that, similar to the global trend, the age-standardized DALY rate of GBTC in the MENA region has been on a downward trend from 1990 to 2021. Additionally, the ASDR of GBTC has decreased globally and has also decreased slightly in the MENA region over the past 32 years, although the decline has not been significant in the region. In contrast, while the ASIR of GBTC has been trending downward globally, it has remained stable with minimal fluctuation in the MENA region. Nonetheless, according to this analysis, the incidence, mortality, and DALY rates of these cancers were lower in this region compared to global rates over the entire period from 1990 to 2021.

GBTC represents a relatively uncommon but highly aggressive malignancy with substantial mortality^[^14^]^. Gallstones are the primary risk factor, present in approximately 80% of cases^[^15,16^]^, while other contributing factors include chronic inflammation, primary sclerosing cholangitis, chronic infections, and lifestyle-related factors such as obesity, diabetes, smoking, and unhealthy dietary habits^[^17^]^. The insidious onset and non-specific early symptoms often result in late-stage diagnosis of GBTC in most patients, leading to a very poor prognosis with a 5-year survival rate of less than 20%^[^18^]^. Over the preceding 32 years, the absolute numbers of incident cases, deaths, and DALYs associated with GBTC have increased markedly^[^3^]^, largely driven by alterations in demographic composition, including an aging population, overall population growth, and shifts in the global geographical distribution of risk factors related to GBTC^[^19,20^]^. However, consistent with the findings of our study in the MENA region, as recently reported in several studies, age-standardized rates of GBTC have been on a downward trend over the past decades globally and in many regions^[^6,21^]^, and are projected to continue decreasing^[^21^]^. Decreased exposure to some modifiable risk factors, influential cancer prevention endeavors, enhanced healthcare infrastructure and medical services, along with public health initiatives and advancements in treatment interventions, may have contributed to the declining trend in age-standardized rates^[^22,23^]^. Although age-standardized rates are generally declining, it is crucial to maintain prevention and reduction efforts for GBTC in MENA, a region that has not experienced the same level of decline as the global average.

The current study also revealed that in both 2021 and 1990, the incidence, mortality, and burden of GBTC increased with age, with the highest rates observed among individuals aged 80 years and older. Previous studies have similarly indicated that older adults are predisposed to a heightened risk of GBTC^[^24–26^]^. Consistent with our findings, a cancer burden analysis in China revealed a markedly increased risk of both incidence and mortality associated with GBTCs among individuals aged 65 years and older, particularly within the 80–94 age range^[^27^]^. While GBTC commonly affects the elderly population, recent studies have indicated a concerning increase in cases of early-onset GBTC among individuals under 50 years of age^[^28^]^. These patients tend to have a more aggressive tumor phenotype and poorer prognostic outcomes^[^29,30^]^. Given these findings, it is imperative to thoroughly evaluate the impact of EOGBM on younger patients and to project its incidence trends over time^[^31^]^. It is crucial to develop prevention strategies and interventions aimed at mitigating the burden of this aggressive malignancy.

Furthermore, across all age groups, the ASIR, ASDR, and age-standardized DALY rates of GBTC were generally higher in females than in males within the MENA region, consistent with global epidemiological evidence^[^32,33^]^. Females are significantly more susceptible to gallstones than males, particularly during their reproductive years^[^34^]^. The female hormone estrogen increases the risk of gallstone formation by raising cholesterol saturation in bile and is known as the main driver behind the higher incidence of gallbladder cancer in females^[^35^]^. Additionally, the higher prevalence of obesity among females contributes to an increased risk of gallstones and GBTC^[^36^]^. An inequality analysis of the 2021 GBD assessed disparities in cancer rates by gender and revealed that, on a global scale, over three decades, females consistently exhibited higher age-standardized DALY rates, even though both the age-standardized DALY rate and ASIR demonstrated continuous declines. In contrast, males experienced a decrease in age-standardized DALY rates but an increase in ASIR, with both metrics ultimately exceeding those of females^[^23^]^. Thus, given the more unfavorable trend, it is essential to also pay more attention to the male elderly population.

We also analyzed heterogeneity and variation in age-standardized GBTC rates among countries in the MENA region. The UAE demonstrated the highest incidence and mortality rates in both 1990 and 2021, followed by Libya and Algeria, while Syria consistently reported the lowest ASIR, ASDR, and age-standardized DALY rate. The higher burden of GBTC observed in the UAE, Libya, and Algeria may be primarily attributed to the high prevalence of gallstone disease, the most significant risk factor for GBTC^[^37,38^]^. The habit of high-calorie food consumption and a Westernized diet, which is increasingly common in high-income Arab Gulf states such as the UAE, promotes cholesterol supersaturation in bile, leading to the formation of gallstones^[^39^]^. Additionally, a sedentary lifestyle contributes to a rising prevalence of overweight, obesity, and related metabolic disorders in the UAE, which have been proven to be associated with various cancers, including GBTC^[^40,41^]^. Additionally, demographic shifts toward older populations due to increasing life expectancy may further contribute to higher incidence rates^[^42^]^. Since the risk of GBTC increases notably with age, a growing population of older individuals may also contribute to a higher incidence of GBTC in the UAE. The high mortality and DALY rates in countries such as Libya and Algeria may reflect disparities in access to healthcare and treatment, a lack of nationwide programs, and inadequate healthcare infrastructure^[^43,44^]^, as well as dietary habits in Libya and Algeria characterized by a high intake of carbohydrates, fatty products, and a low intake of dietary fiber, leading to a high prevalence of gallstone formation and ultimately heightened risk of GBTC^[^45,46^]^.

In Syria, evidence on the prevalence and burden of GBTC is limited. The traditional Syrian diet is characterized by a Mediterranean pattern that is rich in grains, vegetables, and olive oil, while containing lower levels of saturated fats and refined sugars compared to the Gulf diet^[^47^]^. Nevertheless, the prolonged conflict since 2011 has severely shattered the healthcare system and public health surveillance infrastructure^[^48^]^. Therefore, there is a high potential for meaningful under-reporting and under-diagnosis of GBTC cases due to limited access to medical care, demolished facilities, and population displacement. In contrast, Iran has exhibited the most unfavorable trends in the region concerning all age-standardized rates of GBTC, with the most prominent increases observed in ASMR and ASDR, being the sole country with a significant rising trend in ASIR. With the rising rates of obesity, it could be regarded as a major contributing factor to the growing trend of GBTC incidence and burden rates, particularly among women^[^49^]^. Following the increasing prevalence of metabolic syndrome in Iran, there has been a remarkable increase in the incidence of gallstone diseases, raising the risk of GBTC development^[^50^]^.

In contrast, Kuwait and Turkey demonstrated the most significant declining trends in GBTC rates within the region during this period. Kuwait has demonstrated substantial progress in cancer control, which can also be extended to GBTC^[^51^]^. The country has invested significantly in its healthcare system by expanding universal coverage and launching the Kuwait Cancer Center, which focuses on cancer prevention, early diagnosis, and management^[^52^]^. Nationwide initiatives to decrease the modifiable risk factors have been incorporated into comprehensive strategies for disease prevention^[^53^]^. The declining trends in GBTC rates in Turkey can be attributed to effective risk reduction strategies^[^54^]^. The country has implemented robust public health campaigns focused on modifiable risk factors such as smoking cessation, obesity reduction, and enhanced management of metabolic disorders^[^55^]^. Furthermore, Turkey has developed national strategic frameworks for cancer control that encompass the entire continuum of care, from prevention to palliative treatment^[^56,57^]^. These coordinated efforts have likely contributed to the substantial downward trends observed in GBTC rates.

Although this study presents the most recent findings and trends in GBTC burden in the MENA region, it also has several limitations. The data available in the MENA region are highly heterogeneous, making the interpretation of the results at a regional level problematic. There are countries in the MENA region, particularly those affected by conflict, where limited underlying data are available, and the burden estimates reported in the GBD study rely heavily on modeling assumptions, which may reduce the accuracy of the estimates for these countries. Differences in national data registration policies, differences in data collection across the MENA region, and differences in the completeness of registries may affect the reliability of the available epidemiological data. Additionally, political instability in several regions, such as Syria and Yemen, has disrupted the healthcare system, compromising the availability and accuracy of reported data. Thus, the results should be approached with caution, considering potential inconsistencies in the data. Moreover, this study did not evaluate risk factors contributing to GBTC, such as genetic predispositions and lifestyle factors, nor did it assess GBTC types. Further studies are needed to better understand the role of these risk factors and incorporate them into future trend forecasting. This is particularly important to determine the prevalence of modifiable risk factors for GBTC and their contribution to the overall disease burden at country and regional levels, to inform and guide preventive strategies.

## Conclusions

While the burden of GBTC has decreased in the MENA region and has consistently remained lower than the global average, there is considerable heterogeneity across countries in the region that warrants the attention of policymakers. The increasing burden of GBTC in Iran is particularly concerning and highlights the need for studies to explore its underlying causes. Moreover, countries such as the UAE and Libya, which, despite their shared cultural and lifestyle characteristics with other nations in the region, have experienced persistently high burdens throughout the study period, should investigate the potential factors contributing to this trend. Therefore, the findings of this study might have several implications for research and clinical policies in the region. First of all, it identifies populations at higher risk of GBTC in the MENA region, where further studies are needed to determine the causes of these high burdens. Second, these populations might benefit from preventive strategies for the early identification and treatment of the condition.

## Data Availability

We used Global Burden of Disease 2021 data in our study, which is publicly available at https://vizhub.healthdata.org/gbd-results/.
